# NCAM (CD56) Expression in keratin-producing odontogenic cysts: aberrant expression in KCOT

**DOI:** 10.1186/s13005-015-0060-2

**Published:** 2015-02-12

**Authors:** Beatriz Vera-Sirera, Leopoldo Forner-Navarro, Francisco Vera-Sempere

**Affiliations:** Departaments of Stomatology, University of Valencia, Valencia, Spain; Departaments of Pathology, University of Valencia, Valencia, Spain; Service of Pathology, Hospital Universitario y Politécnico La Fe, Avda Campanar 21, Valencia, 46009 Spain

**Keywords:** NCAM, Keratocysts, Keratocystic odontogenic tumor, Orthokeratinized odontogenic cyst, Immunohistochemistry

## Abstract

**Objective:**

To investigate immunohistochemically the expression of neural cell adhesion molecule (NCAM), which has been identified as a signaling receptor with frequent reactivity in ameloblastomas (AB), in a series of keratin-producing odontogenic cysts (KPOCs).

**Material and methods:**

Immunohistochemical expression of NCAM, using a monoclonal antibody, was determined in a series of 58 KPOCs comprising 12 orthokeratinized odontogenic cysts (OOCs) and 46 keratocystic odontogenic tumors (KCOTs), corresponding to 40 non-syndromic KCOT (NS-KCOTs) and 6 syndromic KCOT (S-KCOTs), associated with nevic basocellular syndrome (NBCS).

**Results:**

NCAM expression was negative in all OOCs, but 36.45% of KCOTs exhibited focal and heterogeneous expression at the basal cell level, as well as in basal budding areas and the basal cells of daughter cysts. The latter two locations were especially applicable to S-KCOTs, with focal NCAM reactivity occurring in 66.66% of cases.

**Conclusions:**

Aberrant NCAM expression, in KCOTs but especially in S-KCOTs, together with its immunomorphological location, suggests that this adhesion molecule and signaling receptor plays a role in the pathogenesis of KCOTs, with a probable impact on lesional recurrence.

**Electronic supplementary material:**

The online version of this article (doi:10.1186/s13005-015-0060-2) contains supplementary material, which is available to authorized users.

## Introduction

Keratin-producing odontogenic cysts (KPOCs) form a heterogeneous group of cystic lesions that are often aggressive in character, with high rates of recurrence and multifocality [1]. The lesional spectrum of KPOCs includes odontogenic keratocysts, that according to last World Health Organization (WHO) guidelines [2] are also referred to as keratocystic odontogenic tumors (KCOTs), in accordance with the fact that KCOTs are true tumoral growths. Effective management of these cystic lesions is subject to frequent discussion [3] and malignant transformation is possible, albeit very rare [4].

NCAM (neural cell adhesion molecule), also known as CD56, was originally characterized as a cell-surface glycoprotein member of the immunoglobulin superfamily, implicated in calcium-independent intercellular adhesion [5] and expressed in a wide variety of cells [6]. However, in the past decade the traditional view of NCAM has been challenged, such that it is now also considered a signaling receptor that impacts upon cellular migration, proliferation, differentiation, and survival, thereby playing a role in different models of tumor growth [7].

NCAM immunoreactivity in ameloblastomas within odontogenic tumors has been reported with very high frequency [8-11], and has also been observed in certain ameloblastic carcinomas [9]. In contrast, NCAM reactivity is indicated as relatively infrequent in KCOTs [10,11].

The aim of the present study was to assess the immunohistochemical expression of NCAM in a series of keratin-producing odontogenic cysts (KPOCs), and to evaluate the possible significance of adhesion molecule reactivity in this heterogeneous group of odontogenic cysts.

## Materials and methods

We studied 58 cases of KPOC diagnosed over a period of 10 years at the Department of Pathology of La Fe University Hospital, Valencia, Spain. Histological material was retrieved from storage. Our work formed part of a project previously approved by our Ethics Committee for Biomedical Research (protocol no. 2013/0045).

We selected KPOC cases using a pathological diagnosis database (Pat Win®, ver. 4.1.4), and performed a 10-year retrospective search employing the following search terms: “keratinized cyst”, “keratocyst”, “primordial cyst”, “keratocystic odontogenic tumor (KCOT)”, and “orthokeratinized odontogenic cyst (OOC)”. All of these terms have been used over time to describe various lesions, including KPOCs [4,12].

All original histological sections were reviewed microscopically by two observers, and were reclassified according to the WHO (2005) criteria [2], into 46 examples of KCOT and 12 of OOC. In all KCOT cases the presence or absence of daughter/satellite cysts in the cystic wall, and/or budding areas of the basal layer, was noted because the morphological features of these lesions have been implicated in their recurrence [13,14].

Clinical and radiological data of all patients were collected using the Mizar® (ver. 2.0) medical records platform, in conjunction with the viewfinder software package Luna® (ver. 3.0). Clinical/radiological follow-up findings, the number of recurrences, and any clinical, pathological, or genetic data suggesting syndromic association were retrieved. Following verification of the diagnostic criteria of Kimonis et al. [15] for nevic basocellular syndrome (NBCS), 6 of the 46 KCOT were considered syndromic KCOTs (S-KCOTs). The remaining 40 cases qualified as sporadic or non-syndromic KCOTs (NS-KCOTs). Therefore, the biopsy material finally included was as follows: 12 OOC; 40 NS-KCOT; and 6 S-KCOT.

Sections of 5-μm thickness were cut from the original paraffin-embedded blocks and mounted on poly-L-lysine-coated glass slides prior to immunohistochemical staining, performed using the lyophilized mouse monoclonal antibody NCL-CD56-504 (clone CD564; Novocastra™, Leica Biosystems, Newcastle upon Tyne, UK), at 1/50 dilution, and with a 60-min incubation time. Epitope retrieval proceeded at 97°C for 20 min, in a citrate buffer of pH 9. Immunostaining was visualized using the high-pH EnVision FLEX system (Dako®, Glostrup, Denmark): hematoxylin was used for counterstaining. Tonsil sections served as positive staining controls: the negative controls were mock-stained test sections (the primary antibody was replaced by PBS).

NCAM (CD56) immunostaining was semi-quantitatively assessed on a scale ranging between 0 and 3+ (0 = *absent*; 1+ = *weak*; 2+ = *moderate*; 3+ = *intense*), and also classified as diffuse (>50% of cells), extensive (10-50% of cells) or focal (<10% of cells expressing NCAM).

NCAM immunohistochemical results for the different KPOC subtypes expressed as categorical variables with numbers and percentages, as well as associations between NCAM expression and recurrence, were compared using Firth’s logistic regression test. Statistical analyses were performed using the SPSS for Windows software package (ver. 14.0; SPSS Inc., Chicago, IL). A value of *p* <0.05 was taken to indicate statistical significance.

## Results

Of 58 KPCOs, only 16 cases (27,58%) dysplayed NCAM expresión at the of epithelial level and inmunoreactivity was in positive cases of focal character (<10% of cells expressing NCAM) with a weak (1+) to moderate (2+) intensity, and always demostrating a heterogeneous distribution within the structure of the cystic lesion.

NCAM expression was absent in all 12 OOC cases, with the epithelial lining of the cysts entirely negative for CD56. In contrast, focal NCAM reactivity was observed at the epithelial level in 16 of 46 KCOT cases (36.95%). There was a significant difference in NCAM expression in cases of OOC *vs.* KCOT (*p* = 0.012).

NCAM immunostaining was differentially expressed in NS- and S-KCOTs: four of the six cases of S-KCOT (66.66%) exhibited focal NCAM reactivity at the epithelial level, in contrast to only 12 of the 40 (30%) cases of NS-KCOT (Table 1). OOC did not recur in any case during clinical/radiological follow-up at 39.2 ± 26.01 m. In contrast, all cases of S-KCOT recurred at a mean follow-up of 112 ± 76.1 m. In NS-KCOT0 cases, the recurrence rate was 35% at a mean follow-up of 29.6 ± 31.04 m. When analyses were performed independent of lesional subtype, there was no association between recurrence and NCAM expression (*p* = 0.52).

**Table 1:** **NCAM expression in KPOC**

**Lesional Type**	**No. cases**	**NCAM** ^**+**^ **cases (%)**		**Recurrence**
OOC	12	0/12	(0,00%)	0,00%
S- and NS-KCOT	46	16/46	(36,95%)	43,47%
NS-KCOT	40	12/40	(30,00%)	35,00%
S-KCOT	6	4/6	(66,66%)	100,00%

**Figure 1:**
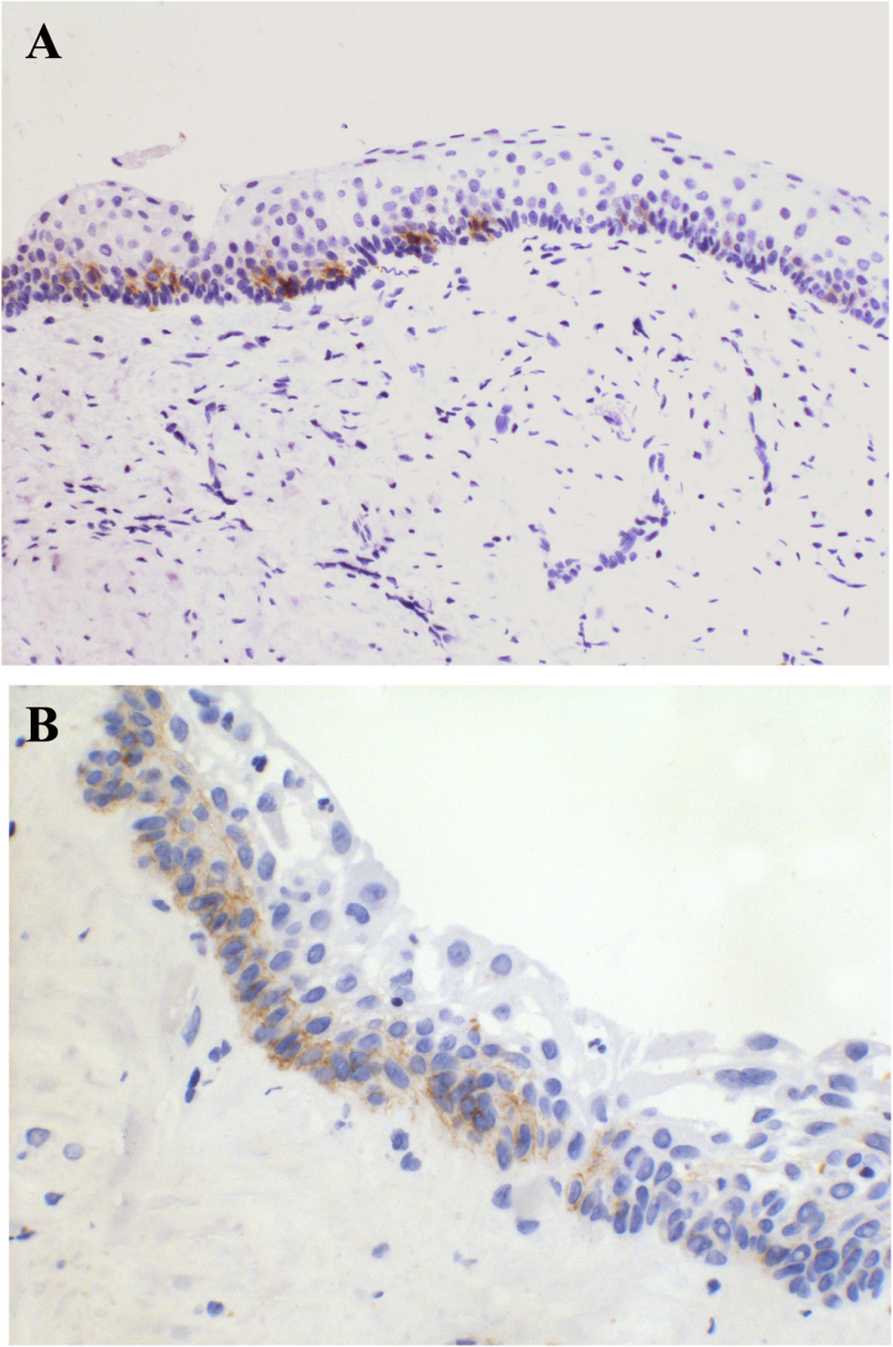
**KCOT showing NCAM reactivity at basal cells with a discontinous pattern (A) and occasional extension to suprabasal level (B).** NCAM 200×.

**Figure 2:**
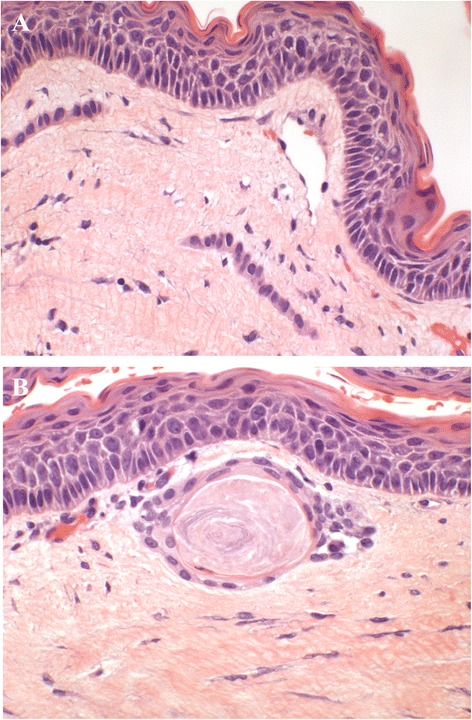
**Epithelial budding (A) and daughter microcyst (B) in KCOT.** HE 200 and 250×.

**Figure 3:**
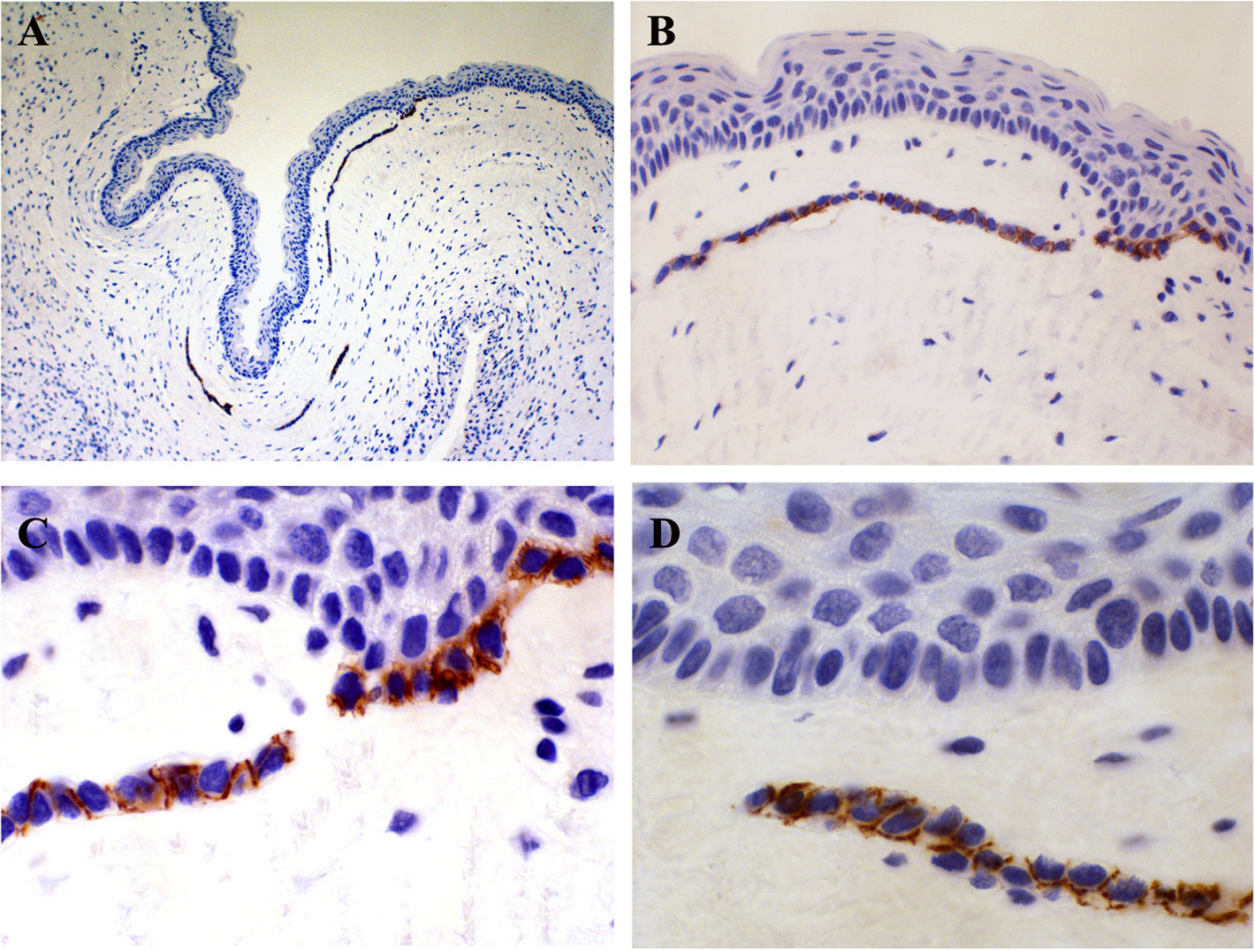
**KCOT showing epithelial buds (A) arising of basal cells (B and C) displaying NCAM reactivity with a predominantly membranous pattern (D).** (NCAM, 200 and 400×).

**Figure 4:**
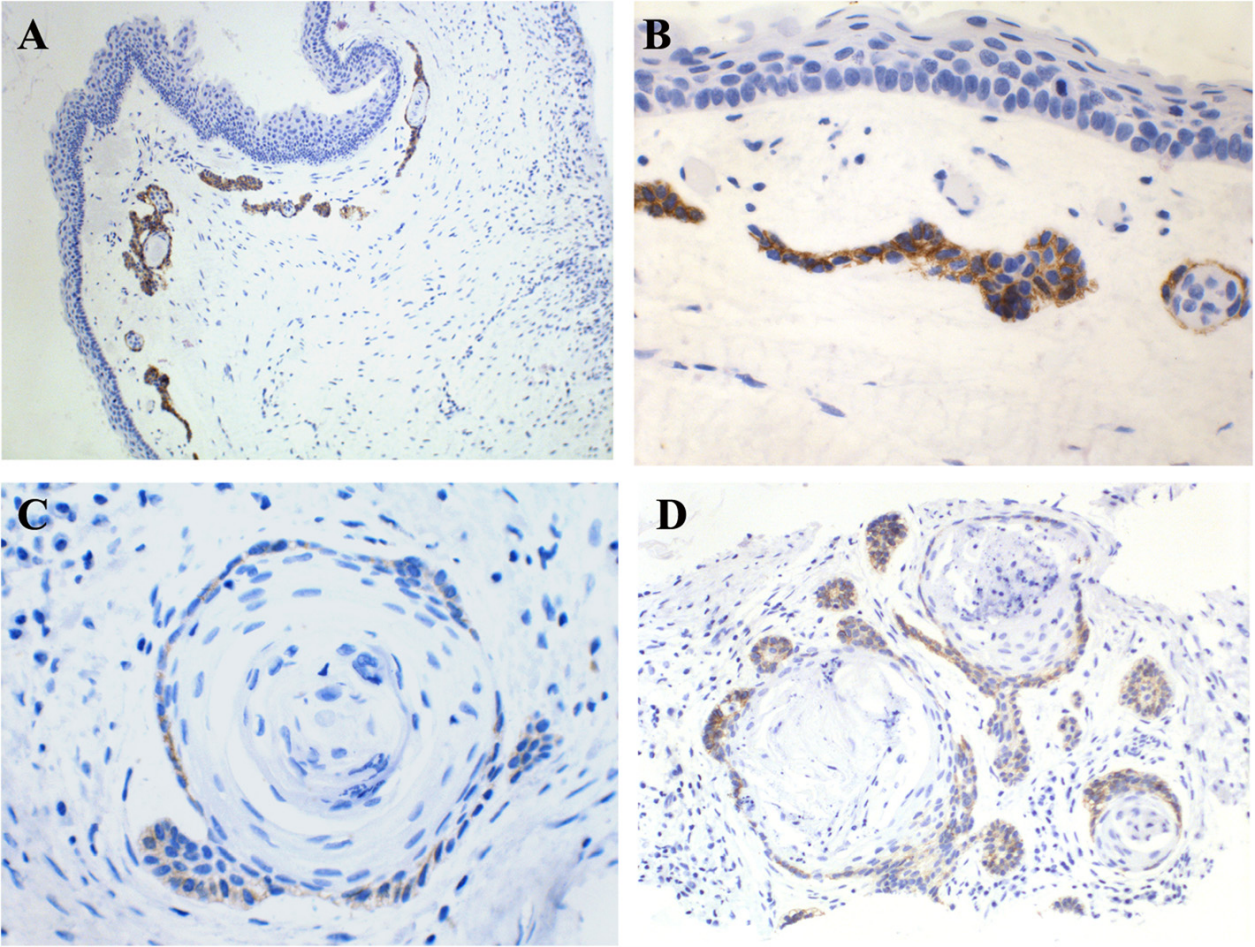
**NCAM reactivity in satellite epithelial nests of small size (A and B) as well as in basal cells of daughter cysts (C and D) located in KCOT wall (NCAM, 200, 250 and 400x).**

In positive cases, NCAM expression was always epithelial, with weak (1+) to moderate (2+) intensity and a heterogeneous and focal distribution (Figures **1**A and **1**B). NCAM positivity (Table 2) was most frequent in the basal layer of cystic epithelium, followed by areas of basal budding (Figures **2**A and **3**A-D) and the basal portion of daughter cysts or satellite epithelial nests (Figures **2**B and **4**A-D). In NCAM-positive S-KCOT cases (four of six), NCAM positivity was discontinuous, weak or moderate, in the locations indicated above, although only 50% of satellite epithelial nests or cysts demonstrated basal positivity. Similarly, in NS-KCOTs, the most frequent NCAM-positive location was the basal cell layer of the cystic lining (observed in all cases exhibiting NCAM positivity), followed by areas of basal budding (*n* = 4) and basal portions of daughter cysts (*n* = 3).

**Table 2:** **NCAM** ^**+**^ **location in KPOC**

**Lesional Type**	**No. cases**	**NCAM** ^**+**^ **cases (%)**		**Basal cells**		**Basal budding**		**Daughter cysts**	
OOC	12	0/12	(0,00%)	0/12	(0,00%)	0/12	(0,00%)	0/12	(0,00%)
S- and NS-KCOT	46	16/46	(36,95%)	16/46	(34,78%)	7/46	(15,21%)	6/46	(13,04%)
NS-KCOT	40	12/40	(30,00%)	12/40	(30,00%)	4/40	(10,00%)	3/40	(7,50%)
S-KCOT	6	4/6	(66,66%)	4/6	(66,66%)	3/6	(50,00%)	3/6	(50,00%)

## Discussion

Neural cell adhesion molecule (NCAM), a member of the immunoglobulin superfamily adhesion molecule, is expressed by a wide variety of neuroectodermal and mesenchymal cells [6]. Originally, NCAM was exclusively characterized as a mediator of cell-cell adhesion, but is now also considered to be a signaling receptor that impacts upon cellular adhesion, migration, proliferation, apoptosis, differentiation, and survival. NCAM is involved in various models of tumorigenesis [7], including certain types of odontogenic tumor: NCAM expression has been reported in certain ameloblastic carcinomas [9], and especially in the outer columnar cells of ameloblastoma (AB) [8-11]. This expression could be indicative of neuroectodermal differentiation in AB [10], especially considering the fact that neural crest cells are associated with tooth development, particularly ectomesenchymal differentiation in tooth germs [15].

The purpose of the present study was to assess the presence and significance of NCAM reactivity in a series of KPOCs. Our results highlight a total absence of NCAM expression in OOCs, a form of KPOCs characterized by orthokeratotic keratinization, reduced proliferative activity and non-recurrence [16,17]. Recurrence was not observed for any case of OOC, but in KCOTs, a form of KPOC now considered as a true odontogenic tumor [1,2,4], we observed focal and heterogeneous NCAM expression in 36.95% of cases, a significantly higher rate than OOC. Prior to our investigation, only two studies assessed NCAM expression in odontogenic cysts. Cairns et al. [11] reported NCAM expression in only 5.26% of KCOTs, with focal NCAM reactivity occurring at the basal cell level; in contrast, AB exhibited high levels of NCAM reactivity. More recently, Kusafuka et al. reported that 50% of KCOTs demonstrated NCAM reactivity, also with exclusive expression at the basal cell level [10]. Our series therefore represented an intermediate rate of positive observations.

In our KCOT cases, NCAM expression was exclusively focal and heterogeneous, with weak-to-moderate reactivity. Despite this limited, focal reactivity, our results demonstrate NCAM expression in locations not described previously: earlier studies noted NCAM reactivity only in the basal cell layer of KCOTs [10,11]. Furthermore, in one of these studies, which analyzed three cases of S-KCOT, there was no relationship between NCAM expression and this syndromic form of KCOT [11]. In contrast, we not only observed discontinuous positivity in the basal portion of cystic epithelium but also in areas of basal budding and in the basal portion of daughter cysts, both of which are occasionally detectable in KCOTs, and particularly in S-KCOTs.

Epithelial budding, arising from the basal layer of cystic lining, is a peculiar morphological aspect of KCOT that has been suggested as the source of daughter cysts; [18-20] influences lesional recurrence; [13,14,21] and is most frequently observed in S-KCOTs [22]. Aberrant NCAM expression in epithelial buds and the basal portion of daughter cysts of KCOTs was observed in 50% of S-KCOTs and represents a possible influence on NCAM expression in instances of lesion recurrence.

In our series, all S-KCOT cases recurred; in NS-KCOTs the recurrence rate was 35%. NCAM expression was detected in 66.66% and 32.50% of S- and NS-KCOTs, respectively. Systematic review of cases of NS-KCOT indicated an average recurrence rate of 28% [23], although rates were heavily influenced by the duration of follow-up, and especially by the type of treatment administered [3], indicating that adequate management of NS-KCOTs may prevent recurrence [2,24].

When we analyzed the relationship between NCAM expression and recurrence independent of KPOC subtype, there was no significant association. However, there was an important limitation: observations were not treated homogeneously due to the retrospective nature of the series. Nevertheless, the fact that NCAM expression was observed in morphological locations involved in recurrence [13,14,21] suggests a possible relationship between NCAM expression and lesional recurrence. Further studies pertaining to NCAM expression, in larger series of KCOTs treated with homogenous procedures or subjected to prospective randomized analysis [25], are required to obtain definitive conclusions. Likewise should be verify a possible association between increased expression of CD56 and others markers [26,27] and genes [28] overexpressed in KCOT.

A final interesting observation concerned NCAM expression in AB, another frequently recurring odontogenic tumor in which NCAM reactivity, in contrast to KCOTs, is virtually certain [11]. AB and KCOTs have been considered two morphologically distinct odontogenic tumors [29]. However, certain morphological and evolutionary data indicate a degree of overlap between these tumors; [30] this similarity is clearly reflected in so-called keratoameloblastoma [31,32], as well as in the solid variant of KCOT [33], characterized by hybrid AB and KCOT morphological features. Accordingly, aberrant NCAM expression in KCOTs could be indicative of foci of ameloblastic differentiation. As suggested previously [27,34], collaborative studies (given the scarcity of observations) concerning NCAM in keratoameloblastomas are required to confirm if this is the case.

## Conclusions

The present study investigated the immunomorphological pattern of NCAM expression in KPOCs, and highlighted that NCAM expression in OOCs is entirely absent. In relation to KCOT our immunohistochemical analysis indicated NCAM reactivity in locations not reported previously (e.g., areas of basal budding and basal cells of daughter cysts), especially with respect to S-KCOTs. This suggests a possible role for this signaling molecule in lesional recurrence in KCOTs. Futures studies using homogeneously treated series of KCOT cases are required to confirm the influence of NCAM on lesion recurrence, as well as its potential utility as a marker of ameloblastic differentiation.

## References

[1] Shear M (2002). The aggressive nature of the odontogenic keratocyst: it is a benign cystic neoplasm? part 1. clinical and early experimental evidence of aggressive behaviour. Oral Oncol.

[2] Barnes L, Eveson JW, Reichart P, Sidransky D (2005). World health organization classification of tumours.

[3] Johnson NR, Batstone MD, Savage NW (2013). Management and recurrence of keratocystic odontogenic tumor: a systematic review. Oral Surg Oral Med Oral Pathol Oral Radiol.

[4] Barghava D, Deshpande A, Pogrel MA (2012). Keratocystic odontogenic tumor (KCOT) - a cyst to a tumour. Oral Maxillofac Surg.

[5] Lanier LL, Chang C, Azuma M, Ruitenberg JJ, Hemperly JJ, Phillips JH (1991). Molecular and functional analysis of human natural killer cell-associated neural cell adhesion molecule (N-CAM / CD56). J Immunol.

[6] Kishimoto T, Kikutani H, von der Borne AEGK, Goyert SM, Mason DY, Miyasaka M (1997). Leukocyte typing VI: white cell differentiation antigens.

[7] Gattenlöhner S, Stühmer T, Leich E, Reinhard M, Etschmann B, Völker HU (2009). Specific detection of CD56 (NCAM) isoforms for the identification of aggressive malignant neoplasms with progressive development. Am J Pathol.

[8] Er N, Dagdevirem A, Tasman F, Zeibek D (2001). Neural cell adhesion molecule and neurothelin expression in human ameloblastoma. J Oral Maxillofac Surg.

[9] Kawai S, Ito E, Yamaguchi A, Eishi E, Okada N (2009). Immunohistochemical characteristics of odontogenic carcinomas: their use in diagnosing and elucidating histognesis. Oral Med Pathol.

[10] Kusafuka K, Hirobe K, Wato M, Tanaka A, Nakajima T (2011). CD56 expression is associated with neuroectodermal differentiation in ameloblastomas: an immunohistochemical evaluation in comparison with odontogenic cystic lesions. Med Mol Morphol.

[11] Cairns L, Naidu A, Robinson CM, Sloan P, Wright JM, Hunter KD (2010). CD56 (NCAM) expression in ameloblastomas and other odontogenic lesions. Histopathology.

[12] Nayak MT, Singh A, Singhvi A, Sharma R (2013). Odontogenic keratocyst: what is the name?. J Nat Sci Biol Med.

[13] Woolgar JA, Rippin JW, Browne RM (1987). A comparative study of the clinical and histological features of recurrent and non-recurrent odontogenic keratocysts. J Oral Pathol.

[14] Kimonis VE, Goldstein AM, Pastakia B (1997). Clinical manifestations in 105 persons with nevoid basal cell carcinoma syndrome. Am J Med Genet.

[15] Obara N, Suzuki Y, Nagai Y, Nishiyama H, Mizoguchi I, Takeda M (2002). Expression of neural cell-adhesion molecule mRNA during mouse molar tooth development. Arch Oral Biol.

[16] Wright JM (1981). The odontogenic keratocyst: orthokeratinized variant. Oral Surg.

[17] Dong Q, Pan S, Sun LS, Li TJ (2010). Orthokeratinized odontogenic cyst. a clinicopathological study of 61 cases. Arch Pathol Lab Med.

[18] Regezi JA (2002). Odontogenic cysts, odontogenic tumors, fibroosseous, and giant cell lesions of the jaws. Mod Pathol.

[19] Kuroyanagi N, Sakuma H, Miyabe S, Machida J, Kaetsu A, Yokoi M (2009). Prognostic factors for keratocystic odontogenic tumor (odontogenic keratocyst): analysis of clinico-pathologic and immunohistochemical findings in cysts treated by enucleation. J Oral Pathol Med.

[20] Mendes RA, Carvalho JFC, van der Waal I (2010). Characterization and management of the keratocystic odontogenic tumor in relation to its histopathological and biological features. Oral Oncol.

[21] Myoung H, Hong SP, Hong SD, Lee JI, Lim CY, Choung PH (2001). Odontogenic keratocyst: review of 256 cases for recurrence and clinicopathological parameters. Oral Surg Oral Med Oral Pathol.

[22] Woolgar JA, Rippin JW, Browne RM (1987). A comparative histological study of odontogenic keratocysts in basal cell naevus syndrome and control patients. J Oral Pathol.

[23] MacDonal-Jankowsk DS (2011). Keratocystic odontogenic tumor: systematic review. Dentomaxillofac Radiol.

[24] Lo Muzio L, Staibano S, Pannone G, Bucci P, Nocini PF, Bucci E (1999). Expression of cell cycle and apoptosis-related proteins in sporadic odontogenic keratocysts and odontogenic keratocysts associated with the nevoid basal cell carcinoma syndrome. J Dent Res.

[25] Zecha JAEM, Mendes RA, Lindeboom VB, Van der Waal I (2010). Recurrence rate of keratocystic odontogenic tumor after conservative surgical treatment without adjunctive therapies – A 35-year single institution experience. Oral Oncol.

[26] Oliveira MG, Lauxen IS, Filho MSA (2005). P53 protein reactivity in odontogenic lesions: an immunohistochemical study. R Fac Odonto Porto Alegre.

[27] Vera-Sirera B, Forner-Navarro L, Vera-Sempere F (2015). Differential expression of cyclin D1 in keratin-producing odontogenic cysts. Med Oral Patol Oral Cir Bucal.

[28] Heikinheimo K, Jee PR, Morgan N, Nagy S, Knuutila S, Leivo I (2007). Genetic changes in sporadic keratocystic odontogenic tumors (odontogenic keratocysts). J Dent Res.

[29] Eversole LR, Sabes WR, Rovin S (1975). Aggressive growth and neoplastic potential of odontogenic cysts: with special reference to central epidermoid and mucoepidermoid carcinomas. Cancer.

[30] Ide F, Ito Y, Muramatsu T, Saito I, Abiko Y (2012). Histogenetic relations between keratoameloblastoma and solid variant of odontogenic keratocyst. Oral Surg Oral Med Oral Pathol Oral Radiol Endod.

[31] Whitt JC, Dunlap CL, Sheets JL, Thompson ML (2007). Keratoameloblastoma: a tumor sui generis or a chimera?. Oral Surg Oral Med Oral Pathol Oral Radiol Endod.

[32] Geng N, Lv D, Chen QM, Zhu ZY, Wu RQ, He ZX (2012). Solid variant of keratocystic odontogenic tumor with ameloblastomatous transformation: a case report and review of the literature. Oral Surg Oral Med Oral Pathol Oral Radiol Endod.

[33] Ketabi MA, Dehghani N, Sadeghi HM, Shams MG, Mohajerani H, Azarsina M (2013). Keratoameloblastoma, a very rare variant of ameloblastoma. J Craniofac Surg.

[34] Geng N, Chem Q-M (2013). Differentiating solid variants of keratocystic odontogenic tumors and keratoameloblastomas. Oral Surg Oral Med Oral Pathol Oral Radiol Endod.

